# Long-term outcome in ICU patients with acute kidney injury treated with renal replacement therapy: a prospective cohort study

**DOI:** 10.1186/s13054-016-1409-z

**Published:** 2016-08-12

**Authors:** Wouter De Corte, Annemieke Dhondt, Raymond Vanholder, Jan De Waele, Johan Decruyenaere, Veerle Sergoyne, Joke Vanhalst, Stefaan Claus, Eric A. J. Hoste

**Affiliations:** 1 Department of Intensive Care Medicine, Ghent University Hospital, De Pintelaan 185, 9000 Gent, Belgium; 2 Department of Anesthesia and Intensive Care Medicine, AZ Groeninge Hospital, Kortrijk, Belgium; 3 Nephrology Division, Ghent University Hospital, Ghent, Belgium; 4 Research Foundation-Flanders (FWO), Brussels, Belgium; 5 Department of Anesthesia, Stedelijk Ziekenhuis, Aalst, Belgium; 6 Department of Anesthesia, Sint-Jozef Ziekenhuis Izegem, Izegem, Belgium

**Keywords:** Acute kidney injury (AKI), Long-term survival, Major adverse kidney events (MAKE), Renal recovery, Renal replacement therapy (RRT), Modality of renal replacement therapy, Timing of renal replacement therapy, Acute-on-chronic kidney failure

## Abstract

**Background:**

In intensive care unit (ICU) patients, acute kidney injury treated with renal replacement therapy (AKI-RRT) is associated with adverse outcomes. The aim of this study was to evaluate variables associated with long-term survival and kidney outcome and to assess the composite endpoint major adverse kidney events (MAKE; defined as death, incomplete kidney recovery, or development of end-stage renal disease treated with RRT) in a cohort of ICU patients with AKI-RRT.

**Methods:**

We conducted a single-center, prospective observational study in a 50-bed ICU tertiary care hospital. During the study period from August 2004 through December 2012, all consecutive adult patients with AKI-RRT were included. Data were prospectively recorded during the patients’ hospital stay and were retrieved from the hospital databases. Data on long-term follow-up were gathered during follow-up consultation or, in the absence of this, by consulting the general physician.

**Results:**

AKI-RRT was reported in 1292 of 23,665 first ICU admissions (5.5 %). Mortality increased from 59.7 % at hospital discharge to 72.1 % at 3 years. A Cox proportional hazards model demonstrated an association of increasing age, severity of illness, and continuous RRT with long-term mortality. Among hospital survivors with reference creatinine measurements, 1-year renal recovery was complete in 48.4 % and incomplete in 32.6 %. Dialysis dependence was reported in 19.0 % and was associated with age, diabetes, chronic kidney disease (CKD), and oliguria at the time of initiation of RRT. MAKE increased from 83.1 % at hospital discharge to 93.7 % at 3 years. Multivariate regression analysis showed no association of classical determinants of outcome (preexisting CKD, timing of initiation of RRT, and RRT modality) with MAKE at 1 year.

**Conclusions:**

Our study demonstrates poor long-term survival after AKI-RRT that was determined mainly by severity of illness and RRT modality at initiation of RRT. Renal recovery is limited, especially in patients with acute-on-chronic kidney disease, making nephrological follow-up imperative. MAKE is associated mainly with variables determining mortality.

**Electronic supplementary material:**

The online version of this article (doi:10.1186/s13054-016-1409-z) contains supplementary material, which is available to authorized users.

## Background

Acute kidney injury (AKI) is a frequent finding in intensive care unit (ICU) patients, with a prevalence of approximately 40–57 % when defined according to the Kidney Disease: Improving Global Outcomes (KDIGO) criteria. AKI treated with renal replacement therapy (AKI-RRT) occurs in approximately 13 % of ICU patients [1, 2]. It is associated with adverse outcomes such as increased length of stay, short- and long-term mortality, and end-stage renal disease (ESRD). In the past, AKI was considered a surrogate marker for severity of illness, and patient mortality was considered a consequence of the underlying disease [3]. However, there is an abundance of epidemiological data demonstrating that AKI in itself leads to adverse outcomes. This is so for the most severe form of AKI, where patients are treated with RRT [4, 5]. In addition, small decreases in kidney function are associated with increased short-term mortality. Further, the prevalence of preexisting chronic kidney disease (CKD) is increasing among patients admitted to the ICU. CKD may lower the threshold for developing AKI, and acute-on-chronic kidney disease is associated with adverse outcomes [3–7]. Further, even mild AKI may predispose patients to CKD, and thus it increases the risk of subsequent AKI events and finally ESRD [8–10]. So, AKI can be considered both the cause and the consequence of CKD, and AKI and CKD therefore are considered interconnected and integrated syndromes [6].

The association of CKD with mortality remains a matter of debate. On one hand, a recent large registry study demonstrated an association of CKD and death [7]. On the other hand, critically ill patients with AKI-RRT who had CKD were reported to have lower short-term mortality than those without preexisting CKD [9, 11–14]. Another factor that may impact long-term outcomes is modality of RRT. Observational studies suggest that continuous RRT (CRRT) is associated with better kidney outcomes, more specifically with less need for chronic dialysis [8, 9]. However, prospective randomized studies could not demonstrate a survival benefit of CRRT compared with intermittent therapies [10, 11]. Finally, optimal timing of initiation of RRT is unclear. RRT is initiated early in the absence of serious complications of AKI and may therefore have some advantages. The late and more conservative approach takes into account that some patients with severe AKI might recover kidney function spontaneously without starting RRT, thereby avoiding adverse events linked to RRT [12].

Until recently, studies of AKI in ICU patients were focused on conventionally accepted short-term outcomes such as mortality at day 30 or at ICU and hospital discharge. However, these endpoints may underestimate the true burden of kidney disease. In light of the increasing focus on long-term outcomes, researchers in several studies have investigated the links between AKI, CKD, and ESRD [13, 14]. By way of analogy to major adverse cardiovascular events, this led to the introduction of the composite endpoint major adverse kidney events (MAKE) [15]. MAKE is a composite of death, ESRD needing dialysis, and incomplete kidney recovery, defined as a 25 % decrease of estimated glomerular filtration rate (eGFR), measured at long-term endpoints such as 90 days or 1 year. The aim of the present study was to describe long-term patient and kidney outcomes in a cohort of patients with AKI-RRT and to assess possible modifying factors of outcome, such as CKD, timing of initiation of RRT, and RRT modality.

## Methods

We conducted a single-center prospective cohort analysis of patients with AKI-RRT at the ICU of the Ghent University Hospital over an 8-year period (October 2004–October 2012). The Ghent University Hospital ICU consists of a 22-bed surgical ICU, a 14-bed medical ICU, an 8-bed cardiac surgery ICU, and a 6-bed burn ICU.

### Study cohort

The inclusion criteria were ICU patients aged ≥15 years who had AKI and were treated with RRT and who had follow-up data after hospital discharge. During the study period, an electronic patient data management system (PDMS) was gradually introduced. Only patients who were registered in the PDMS were included in the study [16]. Exclusion criteria were extracorporeal blood purification techniques for reasons other than AKI, patients with CKD receiving chronic RRT, RRT initiated before admission to the ICU, and RRT immediately after kidney transplant. In cases where a patient had several ICU episodes of AKI-RRT during the same hospital admission, we considered only the first episode.

Indications for RRT, as well as the modality chosen (i.e., intermittent hemodialysis [IHD], duration 2–4 h per treatment session; slow extended daily dialysis [SLEDD], duration 6–12 h per treatment session; or CRRT [continuous venovenous hemofiltration or hemodialysis]), were determined by consensus between the attending intensivists and nephrologists and based on the clinical status of the patient (fluid balance, respiratory status, acid-base balance). Continuous modalities are preferentially used in patients with severe shock, patients who are at risk for cerebral edema (e.g., liver cirrhosis), or patients for whom fluid removal is pursued [17].

### Definitions

Reference serum creatinine was either a baseline serum creatinine concentration obtained from the laboratory database within a 12-month period prior to hospital admission or, if unavailable, serum creatinine at the time of hospital admission. In the latter group, some patients already had AKI at the time of hospital admission. Therefore, in the group for which we had to rely on hospital admission creatinine, we excluded patients who were initiated on RRT within 2 days after hospital admission, as well as patients who had a higher serum creatinine concentration at the time of admission than at hospital discharge. We did not apply back-calculation of baseline serum creatinine with the Modification of Diet in Renal Disease eGFR formula as suggested by the KDIGO AKI guidelines, because this would have led to underestimation of the number of patients with preexisting CKD stage 3 or higher [18].

Timing of initiation of RRT was defined using the KDIGO staging criteria. Initiation of RRT at KDIGO stage 1 or 2 was defined as “early,” and initiation of RRT at KDIGO stage 3 was defined as “late.” Oliguria was defined as diuresis of less than 500 ml over 24 h preceding the initiation of RRT. Fluid balance comprising the 24-h episode before initiation of RRT was calculated by the PDMS. Recovery of kidney function was assessed only in patients with reference creatinine. Recovery of kidney function was classified as complete when eGFR was within 25 % of the reference eGFR (based on reference serum creatinine). Incomplete kidney recovery was defined as patients who had a 25 % or greater decline of reference eGFR and who were not treated with dialysis. Absent kidney recovery was defined as the permanent need for RRT for more than 3 months. Since long-term serum creatinine data were seldom available at the exact follow-up times (e.g., 90 days), we allowed the following intervals: day 90 ± 7 days, 1 year ± 60 days, 2 year ± 60 days, and 3 years ± 60 days.

CKD was defined according to eGFR categories per the KDIGO criteria [19]: Stage 1 CKD is an eGFR >90 ml/minute/1.73 m^2^; stage 2 is 60–90 ml/minute/1.73 m^2^; stage 3 is 30–60 ml/minute/1.73 m^2^; stage 4 is 15–30 ml/minute/1.73 m^2^; and stage 5 is <15 ml/minute/1.73 m^2^ or chronic RRT (hemodialysis or peritoneal dialysis). Patients with CKD stage 3 or worse were classified for the purposes of this study as patients with CKD and compared with patients who had CKD stage 2 or less (no CKD) [15]. Late initiation of RRT was defined as initiation of RRT at KDIGO stage 3. The MAKE composite endpoint was assessed in the patient cohort with reference creatinine, and it was defined as the presence of one or more of the following: death, incomplete kidney recovery, and/or development of ESRD treated with RRT [15].

### Study outcomes

The primary outcome measure of the study was mortality 1 year after initiation of RRT. The secondary outcomes were long-term patient survival and long-term kidney function measured as kidney recovery and dialysis dependence in hospital survivors. In addition, we reported and evaluated the composite outcome measure MAKE. We eventually assessed the classical determinants of long-term outcome of AKI treated with RRT: preexisting CKD, timing of initiation of RRT, and RRT modality.

### Data collection

Data were prospectively recorded during the hospital stay. Baseline demographic parameters were retrieved from the hospital’s electronic database and the ICU’s electronic PDMS. Data on comorbidity and diagnostic categories were retrieved from the hospital administration’s International Classification of Diseases, Ninth Revision, electronic coding system. The severity of illness as determined by the Simplified Acute Physiology Score II (SAPS II) score (based on data recorded during the first 24-h of ICU admission) was recorded at the time of ICU admission [20, 21]. At the time of initiation of RRT, severity of illness was assessed on the basis of parameters of organ dysfunction and Sepsis-related Organ Failure Assessment (SOFA) score [22]. Kidney laboratory data were recorded at hospital admission; ICU admission; initiation of RRT; hospital discharge; and 30 and 90 days and 1, 2, and 3 years. Data on long-term follow-up were gathered from the patients’ electronic medical records (e.g., during follow-up consultation or, in cases of absence of such a consultation, by contacting the primary care physician of the patient by e-mail or telephone).

### Statistical analysis

The data are expressed as number (proportion), median (interquartile range), or OR (95 % CI). Univariate analyses of long-term mortality and MAKE were performed with the Mann-Whitney *U* test, Fisher’s exact test, Friedman’s two-way analysis of variance by ranks test, Wilcoxon rank-sum test, Kruskal-Wallis test, and chi-square test, as appropriate. The predictors thus obtained were subsequently tested in a multivariable logistic regression model. Variables selected for inclusion in the regression model were those with a plausible rationale, with a *P* value ≤0.25 in bivariate analysis. Significant covariates for MAKE were identified after constructing a model in which all covariates were entered simultaneously (enter method). We analyzed for colinearity by assessing correlations between covariates; in addition, interaction was explored. Goodness of fit was assessed according to the method described by Hosmer and Lemeshow. Statistical significance was accepted when the *P* value was <0.05.

The event-free survival rate was estimated using the Kaplan-Meier method, and significance was evaluated with the log-rank test. A Cox proportional hazards model was developed to address the predictors of long-term survival. These analyses were performed with use of IBM SPSS Statistics for Windows, version 23.0.0 (IBM, Armonk, NY, USA).

## Results

**Fig. 1:**
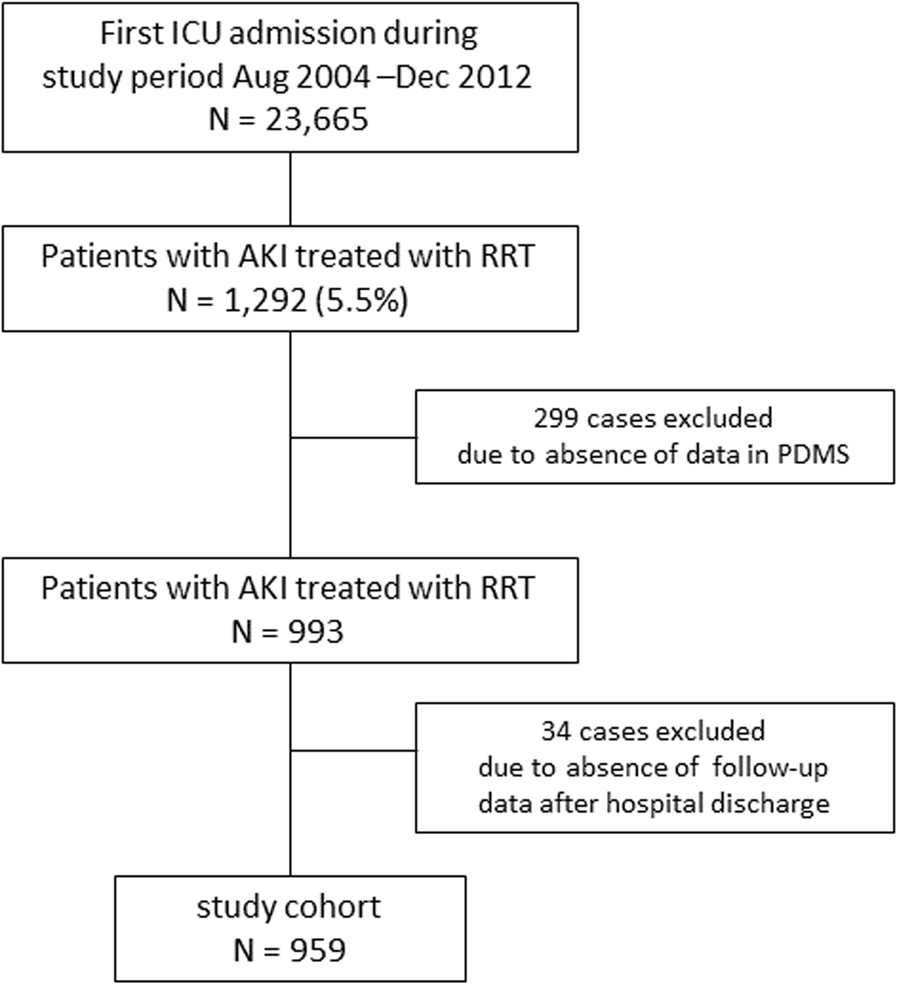
Study flowchart. *PDMS* patient data management system, *AKI* acute kidney injury, *ICU* intensive care unit, *RRT* renal replacement therapy

During the 8-year study period, 23,665 first ICU admissions were registered. A total of 1292 patients (5.5 %) had AKI-RRT, and 959 patients were included in the final analyses (Fig. 1). Of these, 609 patients (63.4 %) had a reference creatinine level documented. Demographic data of the study cohort are shown in Table 1.

**Table 1:** Patient demographics and comparisons

	Total cohort	1-year survivors	1-year nonsurvivors	*P* value	MAKE		*P* value
					1-year absent	1-year present	
Number of patients	959	340	619		102	752	
Demographics							
Age, years	65 (55–75)	64 (52–74)	65 (55–75)	***0.030***	66 (57–74)	65 (55–75)	*0.802*
Male sex, %	65.4	70.0	62.8	***0.026***	64.6	64.0	*0.892*
Black race, %	0.2	0	0.3	*0.294*	0	0.3	*0.593*
Comorbid conditions							
Diabetes mellitus, %	27.5	27.4	27.6	*0.928*	29.4	28.6	*0.864*
Baseline CKD KDIGO stage ≥3, %	39.6	44.2	37.0	*0.084*	51.6	37.3	***0.010***
Baseline eGFR, ml/minute/1.73 m^2^	69.0 (47.0–91.1)	63.4 (44.4–82.9)	74.1 (49.2–93.8)	***0.004***	57.3 (42.8–76.5)	72.9 (49.2–93.4)	***0.001***
Baseline serum creatinine, mg/dl	1.07 (0.82–1.41)	1.15 (0.92–1.48)	1.00 (0.79–1.31)	***<0.001***	1.21 (0.96–1.58)	1.03 (0.80–1.34)	***<0.001***
Characteristics at ICU admission							
Type of ICU							
Medical, %	45.8	36.4	51.1	***<0.001***	32.0	49.6	***0.001***
Surgical, %	54.2	63.6	48.9		68.0	50.4	
Timing of surgery							
Urgent, %	61.0	60.2	61.8	*0.729*	60.2	61.8	*0.729*
Elective, %	39.0	39.8	38.2		39.8	38.2	
Severity of illness							
SAPS II	63 (45–78)	52 (39–69)	70 (54–83)	***<0.001***	49 (34–69)	68 (49–82)	***<0.001***
Characteristics at initiation of RRT							
Severity of illness							
SOFA score, total	10 (6–14)	9 (5–12)	12 (8–15)	***<0.001***	5 (1–9)	11 (7–14)	***<0.001***
SOFA score, nonrenal	7 (3–11)	6 (2–9)	8 (5–12)	***<0.001***	8 (5–12)	8 (4–11)	***<0.001***
Mechanical ventilation, %	88.6	82.4	92.0	***<0.001***	80.8	90.1	***0.006***
Vasoactive medication, %	66.2	52.0	74.0	***<0.001***	48.0	70.4	***<0.001***
Renal characteristics							
Oliguria, %	48.9	45.8	50.8	0.171	36.6	50.9	***0.010***
Fluid balance, ml	2163 (1180–3578)	2021 (1019–3220)	2200 (1268–4000)	***0.049***	1919 (1003–2996)	2229 (1311–4000)	*0.054*
Urine output, ml	561 (178–1080)	621 (246–1230)	529 (157–1020)	*0.055*	792 (297–1483)	529 (152–1055)	***0.007***
Diuretics, %	49.3	56.7	45.2	***0.001***	61.3	45.9	***<0.001***
ICU to RRT length of stay, days	2 (1–7)	2 (1–5)	2 (1–8)	***0.050***	2 (1–6)	3 (1–8)	*0.144*
Serum creatinine, mg/dl	3.57 (2.62–4.69)	4.23 (3.26–5.58)	3.26 (2.38–4.25)	***<0.001***	4.18 (3.11–5.14)	3.38 (2.47–4.44)	***<0.001***
Laboratory parameters							
Serum hemoglobin, g/dl	9.3 (8.3–10.3)	9.5 (8.6–10.5)	9.2 (8.2–10.2)	***0.001***	9.4 (8.6–10.4)	9.2 (8.2–10.2)	***0.001***
Platelets, ×10^3^/mm^3^	115 (67–184)	146 (89–219)	100 (55–158)	***<0.001***	144 (89–202)	104 (60–177)	***<0.001***
Serum sodium, mmol/L	139 (135–144)	138 (134–142)	140 (136–145)	***<0.001***	137 (133–142)	140 (136–144)	***<0.001***
Serum potassium, mmol/L	4.6 (4.1–5.3)	4.8 (4.2–5.4)	4.6 (4.1–5.2)	***0.012***	4.9 (4.2–5.4)	4.6 (4.1–5.2)	***0.007***
Serum chloride, mmol/L	102 (98–107)	101 (97–106)	103 (98–108)	*0.051*	101 (97–106)	103 (98–108)	***0.005***
Serum bilirubin, mg/dl	1.6 (0.7–4.2)	1.3 (0.6–3.3)	1.7 (0.7–4.8)	***0.004***	1.35 (0.60–2.90)	1.27 (0.86–1.82)	***0.001***
Serum urea, g/dl	1.28 (0.90–1.83)	1.33 (0.99–1.83)	1.26 (0.85–1.87)	*0.062*	1.36 (1.00–1.85)	1.27 (0.86–1.82)	*0.173*
Serum albumin, g/dl	2.2 (1.8–2.6)	2.3 (2.0–2.8)	2.1 (1.8–2.5)	***<0.001***	2.3 (2.0–2.6)	2.2 (1.8–2.6)	***0.003***
Lactate, mg/dl	24 (12–82)	15 (10–36)	34 (15–100)	***<0.001***	14 (9–29)	29 (14–94)	***<0.001***
pH	7.30 (7.24–7.37)	7.34 (7.27–7.39)	7.29 (7.21–7.36)	***<0.001***	7.33 (7.27–7.38)	7.29 (7.22–7.36)	***<0.001***
Base excess	−5.2 (−8.3, −2.2)	−4.2 (−6.4, −1.6)	−6.0 (−9.4, −2.5)	***<0.001***	−4.5 (−7.1, −1.8)	−5.5 (−9.1, −2.5)	***0.001***
RRT modality							
IHD, %	54.0	67.6	46.4	***<0.001***	72.5	49.5	***<0.001***
SLEDD, %	15.8	16.2	15.6		14.7	15.5	
CRRT, %	30.2	16.2	38.0		12.7	35.0	
Timing of initiation of RRT							
Late (KDIGO stage ≥3), %	54.1	58.9	51.6	*0.087*	61.1	58.8	*0.735*

*Abbreviations: CKD* chronic kidney disease, *CRRT* continuous renal replacement therapy, *eGFR* estimated glomerular filtration rate, *ICU* intensive care unit, *IHD* intermittent hemodialysis, *KDIGO* Kidney Disease: Improving Global Outcomes, *MAKE* major adverse kidney events, *RRT* renal replacement therapy, *SAPS II* Simplified Acute Physiology Score II, *SLEDD* slow extended daily dialysis, *SOFA* Sepsis-related Organ Failure Assessment

Data are presented as median (interquartile range) unless otherwise indicated

Statistically significant data (*P*<0.05) are presented in bolditalic

### Patient outcome and long-term survival

**Fig. 2:**
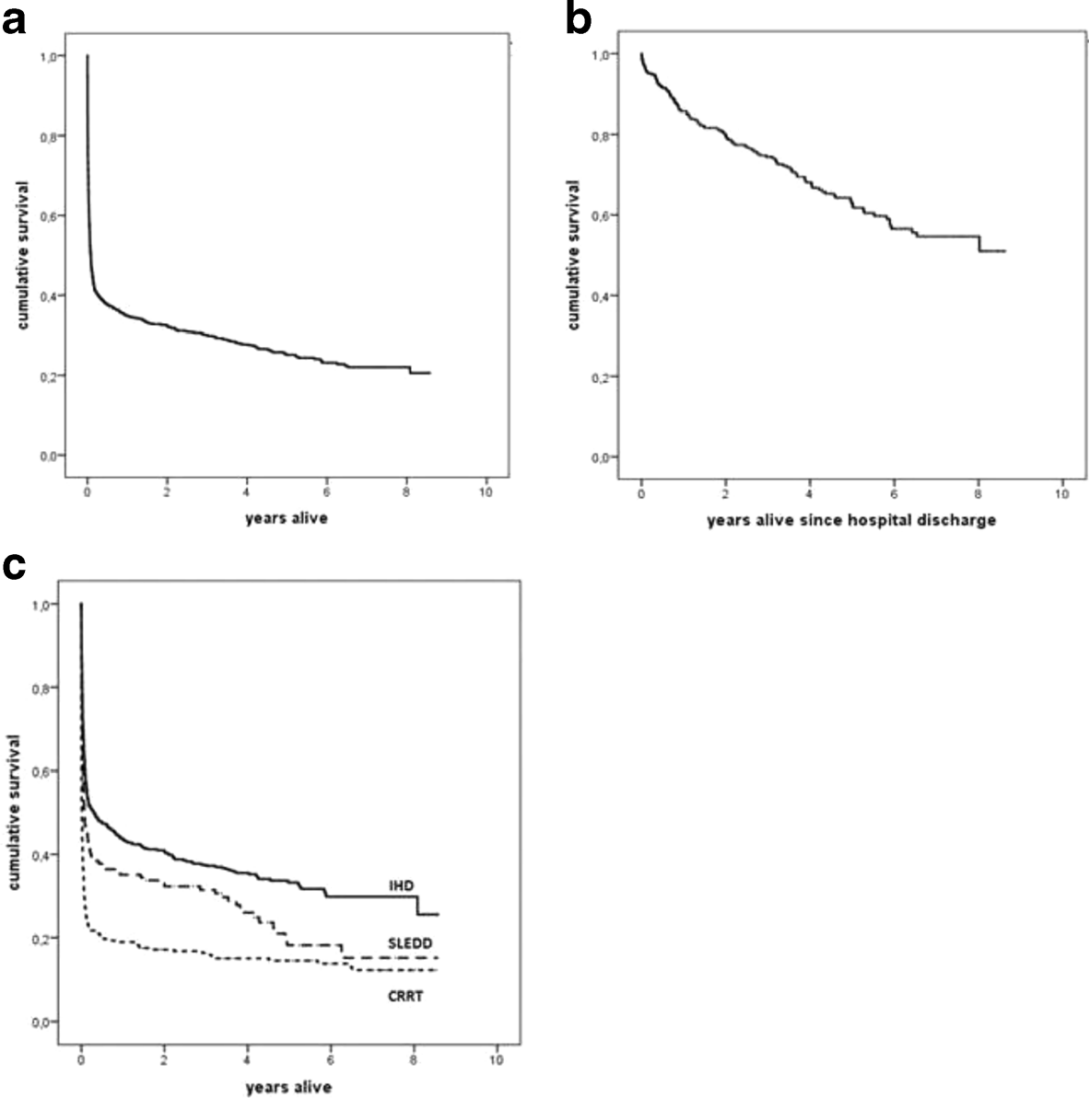
**a** Kaplan-Meier survival curve over time for the whole cohort. **b** Kaplan-Meier survival curve over time for hospital survivors. **c** Kaplan-Meier survival curve stratified for continuous renal replacement therapy (CRRT) modality (*P* < 0.001 by log-rank test), intermittent hemodialysis (IHD), and slow extended daily dialysis (SLEDD)

ICU mortality was 54.6 %. Mortality increased from 59.7 % at the time of hospital discharge to 64.5 % at 1 year, 67.9 % at 2 years, and 72.1 % at 3 years (Fig. 2a). Among hospital survivors, 11.9 % later died at 1 year, 19.3 % at 2 years, and 27.2 % at 3 years (Fig. 2b).

One-year nonsurvivors were significantly older than survivors but had less CKD. A greater proportion of nonsurvivors were female and had been admitted to the medical ICU. At ICU admission, nonsurivors’ severity of illness based on their SAPS II scores were than those of survivors. At initiation of RRT, nonsurvivors had higher SOFA scores than survivors. Their hemodynamic status was more unstable, as a greater proportion were treated with vasoactive agents, had a positive fluid balance, were more acidotic, and had higher serum lactate and more negative base excess. Nonsurvivors were less often treated with diuretics, and a greater proportion were mechanically ventilated and treated with CRRT as the initial RRT modality (Table 1). Patients treated with CRRT as the initial RRT modality had worse survival than patients treated with IHD (*P* < 0.001 by log-rank test) (Fig. 2c).

We found that, after adjustment for confounders in a Cox proportional hazards model, CRRT as the initial RRT modality was associated with long-term mortality (HR 1.570, 95 % CI 1.202–2.050, *P* = 0.001). Baseline kidney function and timing of RRT were not associated with survival in this model. Other confounders associated with survival were older age and increased severity of illness (full model provided in Additional file 1: Table S1).

### Kidney outcomes

Nephrology consultation after hospital discharge was reported in only 34.0 % of hospital survivors. Nephrology follow-up was more frequent in patients with CKD stage ≥3 compared with patients with CKD stage <3 (51.0 % versus 31.8 %, *P* = 0.003). Among hospital survivors, dialysis dependence rates were 8.6 % at hospital discharge, 9.0 % at 90 days, 14.1 % at 1 year, 14.0 % at 2 years, and 16.9 % at 3 years.

**Fig. 3:**
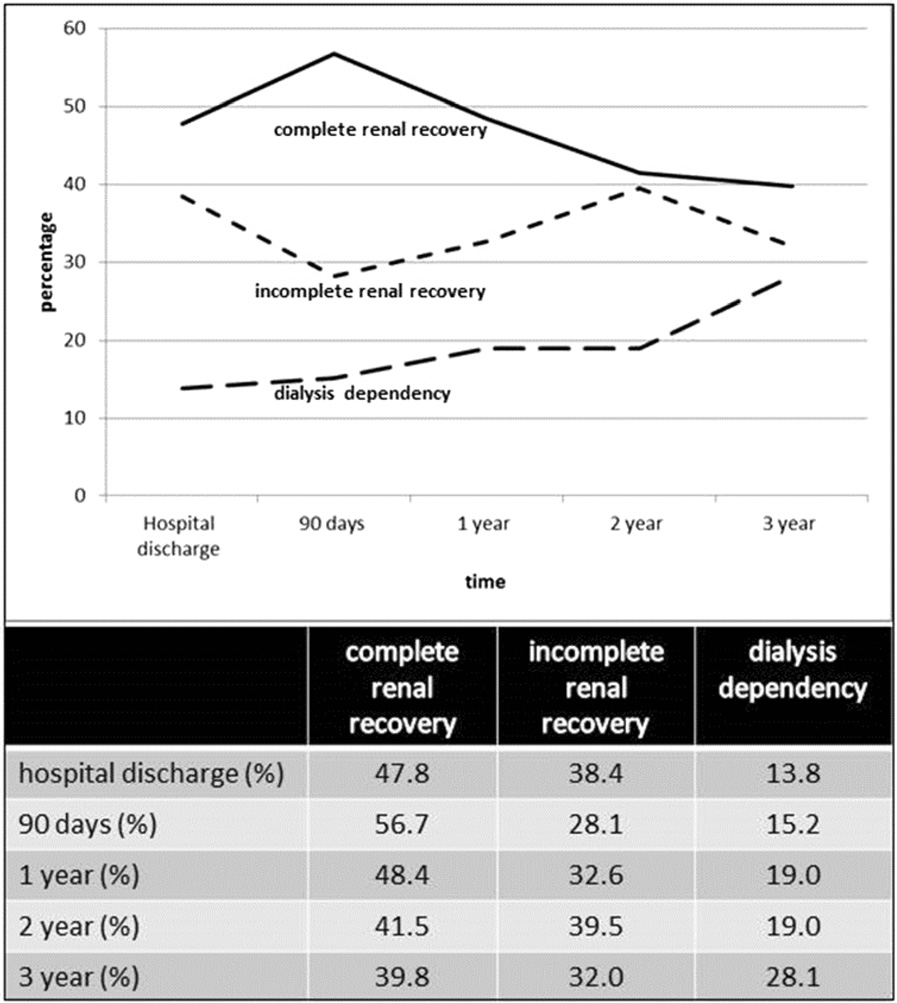
Renal recovery was defined as complete when estimated glomerular filtration rate (eGFR) was within 25 % of baseline eGFR. Incomplete kidney recovery was defined as those patients with an eGFR decrease of 25 % or more from baseline eGFR without need for dialysis. Dialysis dependence was defined as end-stage renal disease and permanent need for renal replacement therapy for >3 months

In order to assess kidney outcomes with a focus on (in)complete renal recovery, the cohort of hospital survivors who had a reference creatinine value was studied (Table 2). In these patients, we found that after 1 year of follow-up, 48.4 % had complete recovery of kidney function, 32.6 % had incomplete recovery, and 19.0 % had ESRD and were being treated with chronic dialysis. Patients who had incomplete recovery had better kidney function and less often had diabetes before AKI. Patients receiving chronic dialysis treatment more often had diabetes, CKD, and oliguria at the time of initiation of RRT. The evolution of kidney outcomes over time are summarized in Fig. 3. Complete renal recovery peaked at 90 days (56.7 %) and further decreased over time. Dialysis dependence increased over time from 13.8 % at hospital discharge to 28.1 % at 3 years. Patients who had prior CKD had more ESRD treated with dialysis than patients without CKD, but they had less incomplete renal recovery (without need for RRT) (Table 3).

**Table 2:** Renal recovery (complete and incomplete) versus dialysis dependence at 1 year in patients with reference serum creatinine values

	Complete renal recovery	Incomplete renal recovery	Dialysis dependence	*P* value
Number of patients (%)	89 (48.4)	60 (32.6)	35 (19.0)	
Demographics				
Age, years (IQR)	67 (57–75)	64 (53–75)	66 (55.75)	*0.682*
Male sex, %	64.0	80.0	57.1	***0.039***
Black race, %	0	0	0	*NA*
Comorbidities				
Diabetes mellitus, %	32.6	21.7	57.1	***0.002***
eGFR, ml/minute/1.73 m^2^	60.6 (43.0–77.2)	73.6 (56.0–95.4)	44.6 (27.1–67.4)	***<0.001***
Baseline CKD stage ≥3, %	49.4	28.3	69.6	***0.013***
Baseline serum creatinine, mg/dl	1.17 (0.94–1.56)	1.05 (0.83–1.29)	1.41 (1.11–2.42)	***0.001***
Characteristics at ICU admission				
Type of ICU				
Medical, %	32.2	35.0	48.5	*0.246*
Surgical, %	67.8	65.0	51.5	
Timing of surgery				
Urgent, %	55.4	64.1	70.6	*0.459*
Elective, %	44.6	35.9	29.4	
Severity of illness				
APACHE II score	25 (19–35)	24 (21–27)	27 (19.31)	*0.948*
SAPS II	50 (35–69)	55 (43–69)	53 (44–71)	*0.871*
Characteristics at initiation of RRT				
Severity of illness				
SOFA score	8 (5–12)	9 (5–12)	5 (5–11)	*0.736*
SOFA score, nonrenal	5 (2–9)	7 (2–9)	2 (1–7)	*0.727*
Mechanical ventilation, %	78.2	85.0	64.5	*0.082*
Vasoactive medication, %	43.7	55.0	35.5	*0.172*
Renal characteristics				
Oliguria, %	39.5	41.5	69.0	***0.019***
Fluid balance, ml	1774 (997–3016)	2546 (1364–3551)	2713 (2103–4534)	*0.059*
Urine output, ml	768 (253–1496)	665 (351–1225)	219 (98–1080)	*0.052*
Diuretics, %	63.2	58.6	40.0	*0.084*
ICU length of stay to initiation of RRT, days	3 (1–4)	3 (2–9)	2 (0–4)	*0.606*
Laboratory parameters				
Serum hemoglobin, g/dl	9.8 (8.9–10.8)	9.3 (8.3–10.5)	9.6 (8.3–10.6)	*0.241*
Serum platelets, ×10^3^/mm^3^	143 (80–195)	164 (87–272)	198 (90–265)	*0.214*
Serum sodium, mmol/L	136 (132–140)	140 (136–142)	139 (133–142)	***0.011***
Serum potassium, mmol/L	5.0 (4.4–5.5)	4.9 (4.2–5.3)	4.7 (4.2–5.1)	*0.264*
Serum chloride, mmol/L	100 (96–105)	102 (97–108)	101 (97–108)	*0.081*
Serum urea, g/dl	1.32 (1.09–1.715)	1.27 (0.92–1.78)	1.34 (1.03–1.97)	*0.664*
Serum creatinine, mg/dl	4.23 (3.27–5.05)	3.95 (3.07–5.08)	4.73 (3.37–6.54)	*0.194*
Serum albumin, g/dl	2.3 (2.0–2.8)	2.3 (1.9–2.6)	2.8 (2.2–3.3)	*0.074*
Lactate, mg/dl	17 (11–33)	16 (11–83)	11 (7–94)	*0.259*
pH	7.34 (7.29–7.39)	7.33 (7.25–7.39)	7.33 (7.24–7.38)	*0.481*
Base excess	−4.0 (−6.6, −1.6)	−4.6 (−6.5, −2.9)	−4.5 (−7.3, −3.3)	*0.628*
RRT modality				
IHD, %	73.0	68.3	*74.3*	*0.799*
SLEDD, %	14.6	16.7	8.6	
CRRT, %	12.4	15.0	17.1	
Timing of initiation of RRT^a^				
Early, %	46.1	35.0	50.0	*0.309*
Late, %	53.9	65.0	50.0	

*Abbreviations: APACHE II* Acute Physiology and Chronic Evaluation II, *CKD* chronic kidney disease, *CRRT* continuous renal replacement therapy, *eGFR* estimated glomerular filtration rate, *ICU* intensive care unit, *IHD* intermittent hemodialysis, *IQR* interquartile range, *KDIGO* Kidney Disease: Improving Global Outcomes, *RRT* renal replacement therapy, *SAPS II* Simplified Acute Physiology Score II, *SLEDD* slow extended daily dialysis, *SOFA* Sepsis-related Organ Failure Assessment

^a^ Early (KDIGO stage <3 at initiation RRT), late (KDIGO stage ≥3 at initiation of RRT

Statistically significant data (*P*<0.05) are presented in bolditalic

**Table 3:** Renal recovery and development of end-stage renal disease in patients with acute-on-chronic kidney disease versus patients without preexisting chronic kidney disease (subgroup analysis in patients with known reference baseline serum creatinine concentration)

Kidney outcome	Total	Preexisting CKD KDIGO stage <3	Preexisting CKD KDIGO stage ≥3	*P* value
Hospital discharge				
Complete renal recovery, %	47.8	47.2	51.3	*0.055*
Incomplete renal recovery, %	38.4	45.7	32.3	
Dialysis dependence, %	13.8	7.1	14.6	
90 days				
Complete renal recovery, %	56.7	55.8	65.2	***0.010***
Incomplete renal recovery, %	28.1	34.6	15.2	
Dialysis dependence, %	15.2	9.6	19.7	
1 year				
Complete renal recovery, %	48.4	47.4	57.1	***0.001***
Incomplete renal recovery, %	32.6	45.3	22.1	
Dialysis dependence, %	19.0	7.4	20.8	
2 years				
Complete renal recovery, %	41.5	34.7	57.1	***<0.001***
Incomplete renal recovery, %	39.5	61.1	22.2	
Dialysis dependence, %	19.0	4.2	20.6	
3 years				
Complete renal recovery, %	39.8	39.1	51.0	***<0.001***
Incomplete renal recovery, %	32.0	51.6	15.7	
Dialysis dependence, %	28.1	9.4	33.3	
MAKE				
Hospital discharge, %	83.1	87.3	51.0	***<0.001***
90 days, %	86.0	81.9	78.2	*0.280*
1 year, %	87.5	87.4	79.4	***0.010***
2 years, %	92.4	92.4	84.2	***0.002***
3 years, %	93.7	92.4	88.5	*0.124*

*Abbreviations: CKD* chronic kidney disease, *KDIGO* Kidney Disease: Improving Global Outcomes, *MAKE* major adverse kidney events

Renal recovery was defined as complete when estimated glomerular filtration rate (eGFR) was within 25 % of baseline eGFR. Incomplete kidney recovery was defined as patients with an eGFR decrease of 25 % or more from baseline eGFR without need for dialysis. Dialysis dependence was defined as end-stage kidney disease and permanent need for renal replacement therapy for >3 months

Statistically significant data (*P*<0.05) are presented in bolditalic

### MAKE

**Fig. 4:**
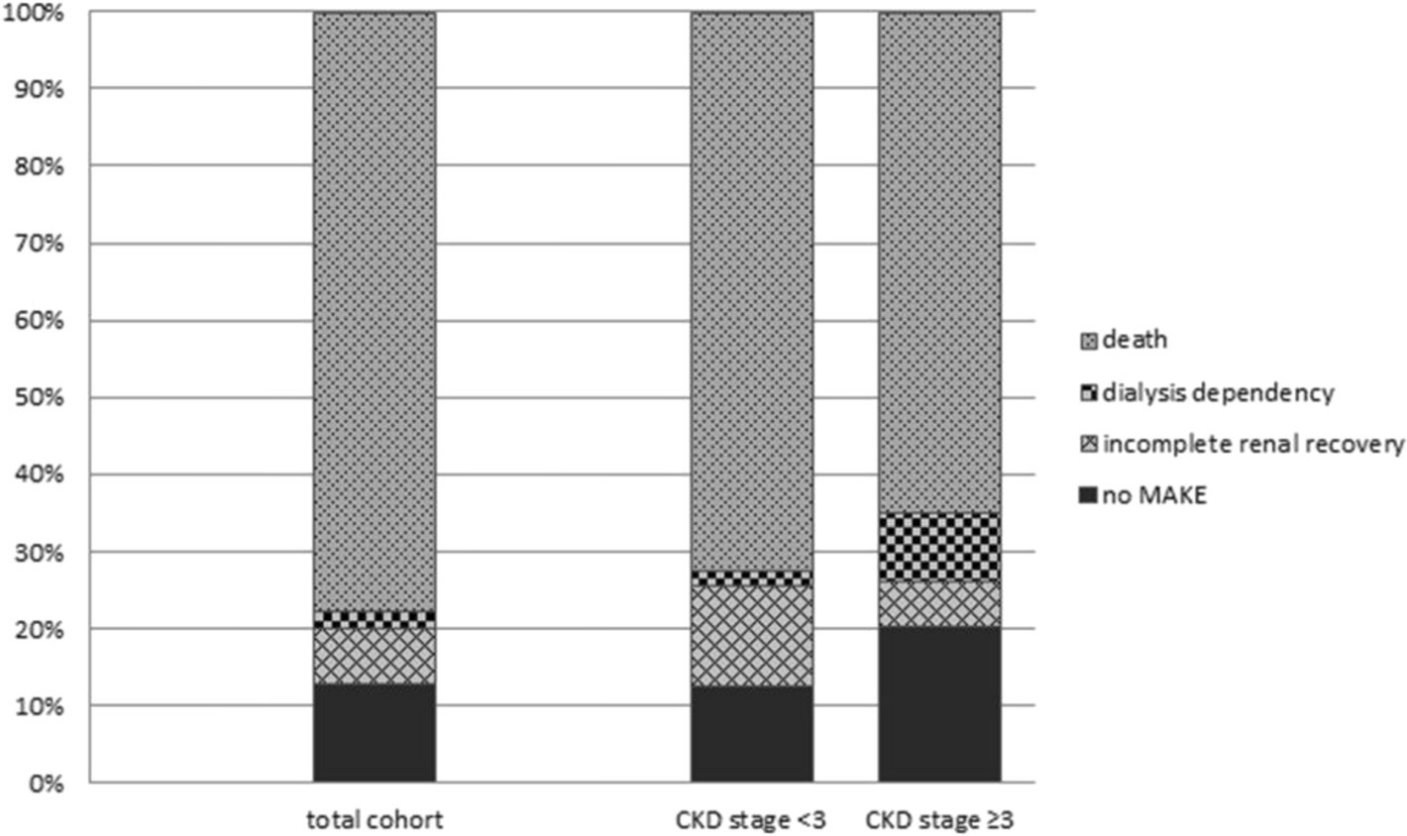
The composite endpoint major adverse kidney events (MAKE) comprised the components death, dialysis dependence, and incomplete renal recovery. Renal recovery was defined as incomplete when estimated glomerular filtration rate (eGFR) decreased 25 % or more from baseline eGFR without need for dialysis. Dialysis dependence was defined as end-stage renal disease and permanent need for renal replacement therapy for >3 months. *CKD* chronic kidney disease

Over time, MAKE increased in the total cohort; it was present in 83.1 % of the patients at hospital discharge, 86.0 % at 90 days, 87.5 % at one year and 92.4 % and 93.7 % at two and three years respectively. MAKE was mainly determined by mortality (Fig. 4). MAKE was more frequent in patients with prior CKD stage <3 compared with patients with preexisting CKD stage ≥3 (Table 3).

### Variables associated with MAKE at 1 year

In univariate analysis, variables associated with MAKE at 1 year were the absence of preexisting CKD, severity of illness on ICU admission and at initiation of RRT (based on SAPS II and SOFA scores, mechanical ventilation, hemodynamic instability with need for vasoactive medication anemia, low platelet count, acidosis, and hyperlactatemia), oliguria, serum creatinine level, and CRRT modality at initiation of RRT (Table 1). On the basis of this univariate analysis, we analyzed associations in a multivariate logistic regression model. After adjustment for confounding covariates, we found that preexisting kidney disease, initial RRT modality, and timing of initiation of RRT were not associated with MAKE at 1 year (full model provided in Additional file 1: Table S2).

## Discussion

We conducted an 8-year analysis of more than 23,000 first ICU admissions and found that AKI-RRT occurred in 5.5 % of patients admitted to the ICU. Mortality rates were high, with almost 60 % of the patients dying during their hospital stay and approximately an additional 10 % per year of the hospital survivors in the years following discharge. Apart from advancing age and increased severity of illness, CRRT as the initial RRT modality was associated with long-term mortality. As for kidney outcomes, almost one-fifth of the AKI-RRT hospital survivors had ESRD at 1 year. Kidney recovery in hospital survivors after AKI-RRT was determined by preexisting renal comorbidity and diabetes mellitus. Finally, after adjustment for covariates, the occurrence of MAKE was not associated with preexisting CKD, timing of initiation of RRT, or RRT modality.

The occurrence rate and mortality of our cohort are concordant with data reported by units in other developed countries [2, 23, 24]. Similarly to other studies, and not surprisingly, long-term mortality was associated with not only advanced age but also variables depicting severity of illness and accompanying hemodynamic instability: use of mechanical ventilation, vasoactive agents, and a positive fluid balance. The association of CRRT as the initial modality of RRT with long-term mortality fits in this concept. In our unit, all modalities are used, and CRRT is used as the initial modality in patients who are in severe shock or for whom slow fluid removal is warranted. When a patient’s condition improves, the modality is switched to SLEDD or IHD. In other words, the choice of the initial modality may serve as a surrogate for severity of illness. Our findings are similar to those of a recent study where RRT modality was also chosen on the basis of the hemodynamic status of the patient [25], but they are in contrast to those in other cohort studies [8, 10, 26, 27]. The recently published studies on timing of RRT by Wald et al, as well as the ELAIN and Artificial Kidney Initiation in Kidney Injury (AKIKI) studies, also illustrate the complexity of the impact of timing on outcomes. While two of these studies could demonstrate no effect of timing, the ELAIN study showed a marked survival benefit for early initiation. Differences between these studies were the definition of early and late initiation, as well as the patients’ characteristics (surgical versus general ICU), modalities used (CRRT in ELAIN versus all modalities in the other studies), and single-center observation (ELAIN) versus multicenter studies (Wald and AKIKI) [28–30].

We found that, among 1-year survivors with known reference serum creatinine values, only 50 % had complete recovery of kidney function. With a dialysis dependence rate of 9.0 % in survivors at day 90, our findings were lower than those reported in the Finnish Acute Kidney Injury (FINNAKI) study (18.9 % at 90 days) and higher than in the Randomized Evaluation of Normal versus Augmented Level Replacement Therapy (RENAL) study (5.6 % at 90 days) and the IVOIRE study (1.4 %) [2, 23, 31]. The FINNAKI, RENAL, and IVOIRE trials used CRRT only, while we started CRRT in only one-fifth of patients.

Interestingly, as dialysis dependence was associated predominantly with comorbidities such as diabetes and CKD, patients with acute-on-chronic kidney disease face a significant risk of developing ESRD. This is similar to findings in other cohort studies and meta-analyses [7, 25, 32, 33].

As many as one-third of patients in our cohort had incomplete renal recovery. Follow-up of patients in the RENAL study also revealed that a large proportion of AKI-RRT survivors had albuminuria and decreased eGFR [34]. Close follow-up and interventions aimed at preserving kidney function may positively impact long-term outcomes. Similar to data reported in the United States [35], only 34.0 % of AKI-RRT survivors in our cohort had follow-up of kidney function by a nephrologist. In our hospital, follow-up by a nephrologist is not protocol-driven but depends on the clinical and renal status of the patient. So, how this possibly impacted kidney outcome and survival is not clear. Especially in patients with acute-on-chronic kidney disease, more standardized kidney follow-up by a general practitioner or nephrologist may be appropriate.

After adjustment for covariates, MAKE was not associated with the classic determinants of outcome, such as preexisting CKD, timing of RRT, or modality of RRT. Our results demonstrate the benefits and limitations of the use of MAKE as a composite endpoint in AKI studies. MAKE is a clearly defined and clinically important endpoint. Compared with single-outcome endpoints, it captures a greater proportion of patients with poor long-term outcomes, turning MAKE into a relevant endpoint. However, detailed evaluation of this outcome parameter necessitates the presentation of the individual components [15, 36–39]. In this study, MAKE was determined mainly by variables associated with its biggest individual component, mortality. Not surprisingly, variables associated with mortality in univariate analysis were also associated with MAKE: increased severity of illness scores and mechanical ventilation but also the presence of hemodynamic instability at initiation of RRT, depicted by the use of vasoactive medication, hyperlactatemia, acidosis, and a positive fluid balance.

This study has several strengths. First, it describes an up to 8-year follow-up period in a large cohort of patients with a heavy burden of disease. Second, apart from the classical mortality rates, we also report detailed information concerning possible determinants of outcome in ICU patients with AKI treated with RRT, such as preexisting CKD, timing of initiation of RRT, and initial modality of RRT. Further, (in)complete renal recovery and dialysis dependence are extensively described. By emphasizing the risk of development of ESRD not only in patients with a single AKI-RRT episode but also in patients with acute-on-chronic kidney disease, this study provides a key role for nephrological follow-up in such a cohort of patients. Finally, this study is one of the first to report on the recently proposed composite endpoint MAKE. The composite endpoint MAKE was addressed in detail, not only revealing its benefits but also highlighting its limitations in this setting. As the study is monocentric, the conclusions cannot automatically be extended to other ICUs. Therefore, generalization of these findings must be considered with caution.

This cohort study has limitations. First, owing to its observational design, we cannot exclude that there were unmeasured confounders. Second, the data reflect the practice at a single tertiary care center and may therefore lack external validity. However, the reported AKI-RRT prevalence of 5.5 % and the hospital mortality rate in this study cohort are in line with data reported by units in other developed countries [2, 27]. Third, we could include only all consecutive patients with AKI-RRT present in the electronic PDMS, owing to its gradual introduction. Similarly, patients who, because of therapeutic restrictions, were not started on RRT were not included in this analysis. Fourth, we had only a reference creatinine value in 63.4 % of patients. Therefore, renal recovery and MAKE were assessed in only a subgroup of patients. Because patients with absent documentation of a baseline serum creatinine level more likely have normal kidney function, this analysis was done in a patient cohort with presumably a higher-than-normal proportion of patients with preexisting CKD. This may have impacted our findings. To correct for possible bias, we performed a sensitivity analysis excluding baseline kidney function from the Cox regression and multivariate analyses. This intervention did not change the HRs and ORs of the covariates included in the model. Therefore, we may conclude that the possibility of bias introduced by this subgroup analysis may be limited.

## Conclusions

We demonstrated poor long-term survival after AKI-RRT associated with advancing age and clinical status at initiation of RRT. Initiation with CRRT, a surrogate for severity of illness, was associated with adverse outcome. Renal recovery was limited and associated with CKD and diabetes. Patients with acute-on-chronic kidney disease frequently developed ESRD, making nephrological follow-up imperative. The majority of patients were classified as MAKE at 1 year. MAKE was determined mainly by its biggest component, mortality. CKD as well as timing and modality of RRT were not associated with MAKE.

## Abbreviations

AKI, acute kidney injury; AKIKI, Artificial Kidney Initiation in Kidney Injury study; AKI-RRT, acute kidney injury treated with renal replacement therapy; APACHE II, Acute Physiology and Chronic Evaluation II; CKD, chronic kidney disease; CRRT, continuous renal replacement therapy; eGFR, estimated glomerular filtration rate; ESRD, end-stage renal disease; FINNAKI, Finnish Acute Kidney Injury study; GFR, glomerular filtration rate; ICU, intensive care unit; IHD, intermittent hemodialysis; IQR, interquartile range; KDIGO, Kidney Disease: Improving Global Outcomes; MAKE, major adverse kidney events; MDRD, Modification of Diet in Renal Disease; PDMS, patient data management system; RENAL, Randomized Evaluation of Normal versus Augmented Level Replacement Therapy study; RRT, renal replacement therapy; SAPS, Simplified Acute Physiology Score; SLEDD, slow extended daily dialysis; SOFA, Sepsis-related Organ Failure Assessment

## Additional file

Additional file 1: Table S1. Cox proportional hazards model. **Table S2.** Multivariate regression analysis: MAKE at 1 year. (DOCX 15 kb)
