# Supplementary material for: Long-term outcome in ICU patients with acute kidney injury treated with renal replacement therapy: a prospective cohort study
Source: Crit Care. 2016 Aug 12;20:256. doi: 10.1186/s13054-016-1409-z (PMC4983760; doi:10.1186/s13054-016-1409-z)
Supplement: Additional file 1: Table S1. — Cox proportional hazards model. Table S2. Multivariate regression analysis: MAKE at 1 year. (DOCX 15 kb) [file 13054_2016_1409_MOESM1_ESM.docx]

Additional electronic files:

Table S1: Cox proportional hazard model

|  | Mortality | | |
| --- | --- | --- | --- |
| Variables | Hazard Ratio | 95% Confidence Interval | *P* |
| Age | 1.021 | 1.011, 1.030 | ***<0.001*** |
| Gender | 1.016 | 0.819, 1.261 | *0.885* |
| GFR baseline | 0.998 | 0.992, 1.005 | *0.573* |
| Admission to MICU | 1.520 | 1.232, 1.875 | ***<0.001*** |
| Ventilation* | 0.928 | 0.646, 1.334 | *0.687* |
| Vasopressor use* | 1.351 | 1.065, 1.713 | ***0.013*** |
| Serum creatinine* | 0.789 | 0.712, 0.875 | ***<0.001*** |
| Serum urea* | 0.993 | 0.962, 1.024 | *0.650* |
| Serum hemoglobin | 1.002 | 1.000,1.004 | *0.112* |
| Blood platelets* | 0.998 | 0.997,0.999 | ***<0.001*** |
| Serum sodium* | 1.036 | 1.016,1.057 | ***<0.001*** |
| Serum potassium* | 1.003 | 0.999,1.008 | *0.158* |
| Serum chloride* | 0.992 | 0.981,1.002 | *0.129* |
| Base Excess* | 0.977 | 0.957,0.997 | ***0.022*** |
| CRRT as initial RRT modality | 1.570 | 1.202,2.050 | ***0.001*** |
| Late initiation of RRT** | 1.279 | 0.943,1.734 | *0.114* |

Legend:

*at initiation of RRT

** KDIGO stage ≥3

GFR (Glomerular Filtration Rate); MICU (Medical ICU); RRT (Renal Replacement Therapy), CRRT (Continuous Renal Replacement Therapy).Table S2: Multivariate regression analysis: MAKE at 1 year

|  | MAKE 1 year | | |
| --- | --- | --- | --- |
| Variables | Odds Ratio | 95% Confidence Interval | *P* |
| Age | 1.029 | 1.003,1.056 | *0.027* |
| Gender | 1.205 | 0.679,2.136 | *0.524* |
| GFR baseline | 1.005 | 0.987, 1.024 | *0.568* |
| Admission to MICU | 1.987 | 1.056,3.737 | ***0.033*** |
| Ventilation* | 0.526 | 0.253,1.096 | *0.860* |
| Vasopressor use* | 1.087 | 0.599,1.974 | *0.783* |
| Oliguria* | 0.958 | 0.537,1.708 | *0.884* |
| Diuretics* | 1.274 | 0.713,2.277 | *0.414* |
| Serum creatinine* | 0.923 | 0.751, 1.133 | *0.444* |
| Serum urea* | 0.919 | 0.641,1.318 | *0.647* |
| Serum hemoglobin | 1.045 | 0.881,1.240 | *0.612* |
| Blood platelets* | 1.000 | 0.997,1.002 | *0.687* |
| Serum sodium* | 1.020 | 0.967,1.077 | *0.464* |
| Serum potassium* | 0.962 | 0.698,1.325 | *0.812* |
| Serum chloride* | 1.003 | 0.974,1.033 | *0.825* |
| pH* | 1.000 | 0.994,1.006 | *0.965* |
| Lactate* | 1.000 | 1.000,1.000 | *0.198* |
| Base Excess* | 1.016 | 0.958,1.078 | *0.593* |
| CRRT as initial RRT modality* | 0.829 | 0.303,2.264 | *0.714* |
| Late initiation of RRT** | 0.763 | 0.348,1.674 | *0.500* |

Legend:

*at initiation of RRT

** KDIGO stage ≥3

GFR (Glomerular Filtration Rate); MICU (Medical ICU); RRT (Renal Replacement Therapy), CRRT (Continuous Renal Replacement Therapy).

Model MAKE 1 year: Goodness of fit (Hosmer and Lemeshow) Chi2 = 4.347, df=8, P= 0.825. Overall percentage correctly predicted = 83.3%.
